# A 16-gene signature predicting prognosis of patients with oral tongue squamous cell carcinoma

**DOI:** 10.7717/peerj.4062

**Published:** 2017-11-17

**Authors:** Zeting Qiu, Wei Sun, Shaowei Gao, Huaqiang Zhou, Wulin Tan, Minghui Cao, Wenqi Huang

**Affiliations:** 1 Department of Anesthesiology, The First Affiliated Hospital of Sun Yat-sen University, Guangzhou, Guangdong, People’s Republic of China; 2 Department of Anesthesiology, Sun Yat-sen Memorial Hospital, Sun Yat-sen University, Guangzhou, Guangdong, People’s Republic of China; 3 Zhongshan School of Medicine, Sun Yat-sen University, Guangzhou, Guangdong, People’s Republic of China

**Keywords:** OTSCC, Oral tongue squamous cell carcinoma, Prognosis, Gene signature, Risk score

## Abstract

**Background:**

Oral tongue squamous cell carcinoma (OTSCC) is the most common subtype of oral cancer. A predictive gene signature is necessary for prognosis of OTSCC.

**Methods:**

Five microarray data sets of OTSCC from the Gene Expression Omnibus (GEO) and one data set from The Cancer Genome Atlas (TCGA) were obtained. Differentially expressed genes (DEGs) of GEO data sets were identified by integrated analysis. The DEGs associated with prognosis were screened in the TCGA data set by univariate survival analysis to obtain a gene signature. A risk score was calculated as the summation of weighted expression levels with coefficients by Cox analysis. The signature was used to distinguish carcinoma, estimated by receiver operator characteristic curves and the area under the curve (AUC). All were validated in the GEO and TCGA data sets.

**Results:**

Integrated analysis of GEO data sets revealed 300 DEGs. A 16-gene signature and a risk score were developed after survival analysis. The risk score was effective to stratify patients into high-risk and low-risk groups in the TCGA data set (*P* < 0.001). The 16-gene signature was valid to distinguish the carcinoma from normal samples (AUC 0.872, *P* < 0.001).

**Discussion:**

We identified a useful 16-gene signature for prognosis of OTSCC patients, which could be applied to clinical practice. Further studies were needed to prove the findings.

## Introduction

Based on GLOBOCAN estimates, cancers of the lip and oral cavity affected about 300,373 new cases and killed about 145,353 people all over the world in 2012 (Torre et al., 2015). Oral tongue squamous cell carcinoma (OTSCC) is a tongue-derived oral cavity squamous cell carcinoma (OCSCC). According to the tumor node metastases (TNM) staging system of the American Joint Committee on Cancer (AJCC), OTSCC can be classified into stage I–IV (Edge, 2010). Either primary surgery or definitive radiation therapy is optional for stage I and II early OTSCC patients (Fujita et al., 1996; Hicks Jr et al., 1998). As for stage III and IV advanced OTSCC patients, surgery plus postoperative radiation therapy or chemoradiotherapy is recommended (Fein et al., 1994; Sessions et al., 2002). The five-year survival rate was 67% for the AJCC stage I, and 51% for the AJCC stage II. The five-year disease-specific survival rate was 39% for stage III, and 27% for stage IV (Rusthoven et al., 2008; Sessions et al., 2003). However, even among patients with the same TNM staging, the prognosis may be different from each other. Therefore, in order to predict prognosis of patients with OTSCC precisely, there is an urgent need to discover potential molecular prognostic biomarkers.

Recently researchers have indicated that some biomarkers served as molecular prognostic markers of OTSCC. For example, MTUS1 (microtubule associated scaffold protein 1) was found to play major roles in the progression of OTSCC, and down-regulation of MTUS1 was associated with reduced overall survival (Ding et al., 2012). Overexpression of PARVB (parvin beta) increased cell migration capability and forecasted poor metastasis-free survival in OTSCC (Eslami et al., 2015). Overexpression of long non-coding RNA (lncRNA) LINC00673 promoted invasion and metastasis, and presented poor prognosis in OTSCC (Yu et al., 2017). MicroRNA miR-26a and lncRNA MEG3 (maternally expressed 3) was reported to have an antitumor effect, and reduced miR-26a and MEG3 was also associated with poor prognostic outcomes (Jia et al., 2014). Meanwhile, gene signatures have been widely used for prognosis of cancers (Shi & He, 2016; Wang et al., 2016; Zhan et al., 2015). When it comes to OTSCC, Krishnan identified a 38-gene minimal signature by machine-learning method, which could predict tumor recurrence (Krishnan et al., 2015). Another DNA methylation signature was established usinggenome-wide methylation analysis, and it was involved with risk habits, clinical, and epidemiologic outcomes (Krishnan et al., 2016). However, researches about gene signatures focusing on the overall survival of OTSCC are limited and it needs further study.

In this study, we obtained five mRNA expression profiling microarray data sets from the Gene Expression Omnibus (GEO, http://www.ncbi.nlm.nih.gov/geo/) and another mRNA sequencing (mRNA-seq) data set from The Cancer Genome Atlas (TCGA, https://cancergenome.nih.gov/). Then, we built a gene signature for prognosis of OTSCC patients by significance analysis of gene expression profiles and Cox regression survival analysis. The gene signature may be meaningful and credible to illuminate the pathogenic mechanism of OTSCC, which could be applied to clinical practice.

## Materials & Methods

### The GEO data sets and integrated analysis

We downloaded five gene expression data sets from GEO database, including GSE2280, GSE3524, GSE6631, GSE9844 and GSE31056. The online tool NetworkAnalyst (http://www.networkanalyst.ca/) was adopted for analysis of annotation from probesets to genes, quantile normalization, gene expression profiling and differentially expressed gene (DEG) identification (Xia, Gill & Hancock, 2015). Additionally, integrated analysis of DEGs across the five GEO data sets was performed by Fisher’s method, which combined the adjusted *P* value. DEGs were selected significantly with the criterion of combined adjusted *P* < 0.05 (Tang & Zhang, 2016). All the default parameters were chosen. The batch effect across different data sets were checked and adjusted online by NetworkAnalyst.

### Enrichment analysis and protein-protein interactions

Gene Ontology (GO, http://geneontology.org) offers a biological model classifying gene functions into the biological process, molecular function and cellular component (Ashburner et al., 2000). The Kyoto Encyclopedia of Genes and Genomes (KEGG, http://www.genome/ad.jp/kegg/) is a database about genomes, biological pathways, diseases, drugs, and chemical substances (Ogata et al., 1999). In this study, GO annotation analysis and KEGG pathway enrichment analysis of DEGs were performed using the Database for Annotation Visualization and Integrated Discovery (DAVID, https://david.ncifcrf.gov/) (Dennis Jr et al., 2003). The *P* < 0.05 and gene counts >2 were considered significant. The Search Tool for the Retrieval of Interaction Genes/Proteins (String, http://string-db.org/) database provides a critical assessment and integration of protein-protein interaction (PPI) based on the DEGs (Szklarczyk et al., 2017). After that, the PPI network was re-constructed with Cytoscape (http://www.cytoscape.org/) software. Since nodes with high connectivity degree contribute more to the stability of the network, we calculated the connectivity degree of each protein node in the PPI network and identified the top five as the hub nodes using the Cytoscape plugin NetworkAnalyzer. Then, the whole significant genes were clustered into several groups to dig out the important cluster using the Cytoscape plugin MCODE.

### The TCGA data set and screening process

By using the R package TCGA-Assembler Version 2.0 (Zhu, Qiu & Ji, 2014), we obtained whole genome mRNA-seq expression data of head and neck squamous cell carcinoma (HNSCC) from the TCGA database (Zhu, Qiu & Ji, 2014). Clinical data were also downloaded through TCGA-Assembler. Patients with OTSCC were extracted with ICD-O-3 (International Classification of Diseases for Oncology, Third Edition) code of C01.9, C02.0, C02.1, C02.2, C02.3, C02.4, C02.5, C02.6, C02.7, C02.8, C02.9. Moreover, histological types were limited to squamous cell carcinoma (code 8050, 8051, 8052, 8070, 8071, 8072, 8073, 8074, 8075, 8076, 8081, 8082, 8083 and 8084). Genes with the expression of zero across all the patients were omitted. Patients with missing survival data were excluded. Quantile normalization and expression calculation of the mRNA-seq data was performed by the R package DESeq ( Anders &Huber, 2010).

During the screening process, for one certain gene, each patient was classified into the high or low expression group by the cutoff of the gene expression median value. Taking the overall survival outcome and survival time into account, we used the univariate Kaplan–Meier analysis to find the association between the certain gene and the survival outcome. Applying it to all the DEGs, the whole survival related genes were constructed (An et al., 2015; Kanth et al., 2016; Xu et al., 2017).

### The gene expression signature and risk score

The gene expression signature was made up of genes associated with clinical survivals. For each patient, the risk score was calculated by the summation of the mRNA expression intensities weighted by corresponding coefficients, which were derived from univariate Cox regression analysis associated with survival outcomes as follows: Risk score =β_gene1_ × expression-value_gene1_ + β_gene2_ × expression-value_gene2_ + ⋯ + β_geneN_ × expression-value_geneN_.

The larger the score, the higher the risk of death outcomes. Consequently, the patients were divided into high-risk and low-risk groups by the median of risk scores. In addition, we utilized the gene expression signature to distinguish carcinoma and normal samples by multivariate logistic regression analysis. Receiver operator characteristic (ROC) curves were employed to detect the classification performance of 16-gene signature by assessing accuracies and specificities. Logistic regression analysis was calculated using R package stats ( R Core Team, 2016). ROC and area under the curve (AUC) were estimated using the R packages pROC ( Robin et al., 2011) and Epi ( Carstensen et al., 2017).

In order to prevent overfitting problems, cross-validation was also performed for validation in TCGA and GEO data sets. For the TCGA data set with survival information, visual calibration curves and concordance indices (C-index) were created to evaluate the performance and predicting ability of the risk score by R packages of rms. Bootstrap with 1,000 resamples and 2-fold cross-validation was set. As for the GEO data sets, 10-fold cross-validation was chosen to assess the classification performance of the gene signature with the R package caret ( Kuhn, 2008).

### Statistical analysis

All the data analysis in this study was conducted with R version 3.3 ( R Core Team, 2016) along with an open source software for bioinformatics called Bioconductor version 3.3 (http://bioconductor.org/). We described continuous variables as means and standard deviations and described categorical variables as frequencies and percentages. For categorical variables, we chose the Pearson’s chi-squared test and Fisher’s exact tests to detect the statistical difference. For continuous variables, we chose independent Student’s *t*-test and Analysis of Variance. When homogeneity of variance did not correspond, nonparametric test of Kruskal–Wallis test was adopted. We selected Kaplan–Meier analysis, univariate and multivariate Cox regression models to distinguish risk factors for overall survival (OS) with R packages KMsurv ( Klein &Moeschberger, 1997) and Survival ( Therneau, 2015). For OS analysis, any cause of deaths was defined as events and survivors were defined as censored events. All *P* values were two-sided and *P* < 0.05 was considered significant.

## Results

### Overview of workflow

**Figure 1:**
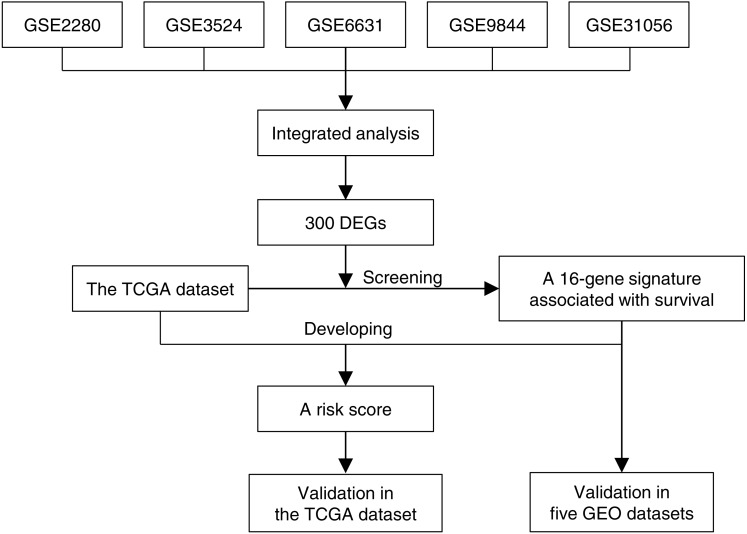
The 16-gene signature development and validation workflow.

Figure 1 illustrated the overview of 16-gene signature development and validation workflow. Five GEO gene expression data sets of OTSCC were annotated, normalized and integrated. Gene expression profiles were compared between tongue carcinoma and normal samples for recognition of DEGs. Next we screened these DEGs in the TCGA data set along with survival information, and found a 16-gene signature associated with survival. Based on the TCGA data set and the 16-gene signature, we developed a risk score, which stratified patients into high-risk and low-risk groups. The risk score for prognosis was verified to be effective in the TCGA data set by univariate and multivariate survival analysis. Additionally, we even exhibited the effectiveness of 16-gene signature to classify the carcinoma samples in the five GEO data sets, which was evaluated based on the ROC curve and AUC.

### The integrated analysis of five GEO data sets

The characteristics of GEO data sets in the integrated analysis were presented in Table 1. We totally included 60 carcinoma samples and 31 control samples from five GEO data sets. With the criterion of combined *P* < 0.05, we identified 300 DEGs when comparing carcinoma with normal samples. As Table S1 showed, the top five significant GO biological process terms of DEGs were SRP-dependent cotranslational protein targeting to membrane, viral transcription, translational initiation, nuclear-transcribed mRNA catabolic process, nonsense-mediated decay and rRNA processing. Table S2 showed that the top five significant KEGG pathways of DEGs enriched in ribosome, viral myocarditis, protein export, lysosome and natural killer cell mediated cytotoxicity. With the medium confidence of 0.400, 297 nodes (protein) and 1,223 edges (interaction) were included in the PPI network based on String database, as shown in Fig. S1. Topological analysis by plugin NetworkAnalyzer identified several ribosomal proteins (RP) as hub nodes in the whole network, including RPL12, RPS11, RPL24, RPS12 and RPS6. As Table S3 demonstrated, three modules were recognized with a score >4 by plugin MCODE as significant clusters in the PPI network.

**Table 1:** Characteristics of five GEO datasets in the integrated analysis.

Dataset Series	Number of Samples		Platform
	Carcinoma	Control	
GSE2280	14	2	Affymetrix Human Genome U133A Array
GSE3524	6	2	Affymetrix Human Genome U133A Array
GSE6631	3	3	Affymetrix Human Genome U95 Version 2 Array
GSE9844	26	12	Affymetrix Human Genome U133 Plus 2.0 Array
GSE31056	11	12	Affymetrix GeneChip Human Genome HG-U133 Plus 2 Array

**Notes.**

GEO Gene Expression Omnibus

### Characteristics of the TCGA data set

The TCGA mRNA-seq expression data set comprised 20,531 genes from 555 patients diagnosed with HNSCC. After excluding 401 genes with zero expression level across all the patients, as well as including 101 OTSCC patients with clinical survival data, we finally got a normalized expression matrix of 20,130 genes from 101 OTSCC patients, including 69 males and 32 females. There were 88 white people and 13 others. Fifty patients were older than 60 years old while 51 patients were less than 60. Among them, 76 patients were diagnosed with G1/2, while 20 with G3/4. There were 28 patients with stage I/II and 69 patients with stage III/IV respectively. The median follow-up period was 701 days (ranging from 64 to 5,480 days).

### The 16-gene signature and risk score development

We aimed at the 300 DEGs from the above-mentioned GEO integrated analysis, and we screened the relationship between the expression levels of those genes and clinical OS in the TCGA data set. It was revealed that the 16 genes were independent prognostic risk factors for OS significantly (*P* < 0.05) after the screening process. They consisted of CD69 (CD69 molecule), CDS2 (CDP-diacylglycerol synthase 2), CPE (carboxypeptidase E), EVI2A (ecotropic viral integration site 2A), FAM69A (family with sequence similarity 69 member A), GUSB (glucuronidase beta), HNF1B (HNF1 homeobox B), ITM2A (integral membrane protein 2A), MBD4 (methyl-CpG binding domain 4), NPY (neuropeptide Y), RGS5 (regulator of G protein signaling 5), SEL1L3 (SEL1L family member 3), SELL (selectin L), SMG1 (nonsense mediated mRNA decay associated PI3K related kinase), SNX4 (sorting nexin 4) and ZC3H3 (zinc finger CCCH-type containing 3), which built up the 16-gene signature. Among them, HNF1B, NPY, SMG1, ZC3H3 were shown to be protective factors, while the others were risk factors. The 16-gene signature was of significance for prognosis for OTSCC. Based on the expression levels of these 16 genes as well as the OS data, we set up the risk score for each patient, which was the weighted sum of the 16-gene expression quantity. The coefficients for the 16-gene signature were displayed as Table 2. The higher risk score represented worse clinical prognosis. Consequently, the risk score stratified the whole patients into two groups by the cut-off of median.

**Table 2:** Coefficients of the 16-gene signature for the risk score.

Gene Symbol	Entrez ID	Coefficient	HR	95% CI	*P* Value
CD69	969	0.984	2.674	1.168–6.122	0.020
CDS2	8760	1.369	3.930	1.626–9.500	0.002
CPE	1363	0.793	2.211	0.997–4.900	0.051
EVI2A	2123	0.831	2.295	1.018–5.174	0.045
FAM69A	388650	0.844	2.325	1.033–5.235	0.041
GUSB	2990	0.987	2.682	1.179–6.102	0.019
HNF1B	6928	−0.917	0.400	0.184–0.868	0.020
ITM2A	9452	0.812	2.252	1.009–5.026	0.047
MBD4	8930	0.915	2.497	1.126–5.537	0.024
NPY	4852	−0.897	0.408	0.190–0.876	0.021
RGS5	8490	0.952	2.591	1.138–5.898	0.023
SEL1L3	23231	0.859	2.360	1.063–5.238	0.035
SELL	6402	0.946	2.574	1.124–5.896	0.025
SMG1	23049	−0.974	0.378	0.163–0.875	0.023
SNX4	8723	1.226	3.408	1.445–8.039	0.005
ZC3H3	23144	−0.962	0.382	0.172–0.848	0.018

**Notes.**

HR hazard ratio CI confidence interval

### Validation in TCGA and GEO data sets

**Figure 2:**
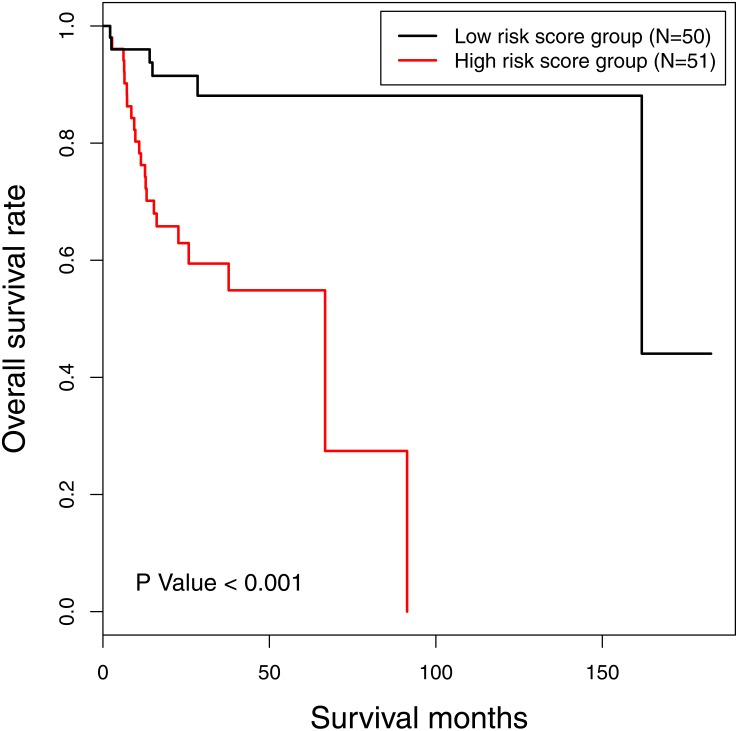
The Kaplan-Meier survival curve: the overall survival in patients with oral tongue squamous cell carcinoma according to risk score. *χ*^2^ = 14.6, *P* < 0.001.

The risk score of 16-gene signature was subsequently validated in the TCGA data set. Every patient was allocated into a high-risk score or low-risk score group, and univariate analysis discovered the risk score as a prognostic factor associated with OS significantly (*P* < 0.001) (Fig. 2). Besides, we included clinicopathological features in the multivariate analysis, and found the risk score remaining as an independent prognostic predictor for OS (HR [hazard ratio] 5.782, 95% CI [2.058–16.244], *P* < 0.001). The calibration curves moved towards the 45-degree straight line passing through the origin, displaying an exceptional performance of the risk score in predicting the 3-year and 5-year OS probabilities (Fig. S2). The C-index predicting OS was 0.652 (95% CI [0.549–0.754]) corrected as 0.654.

**Figure 3:**
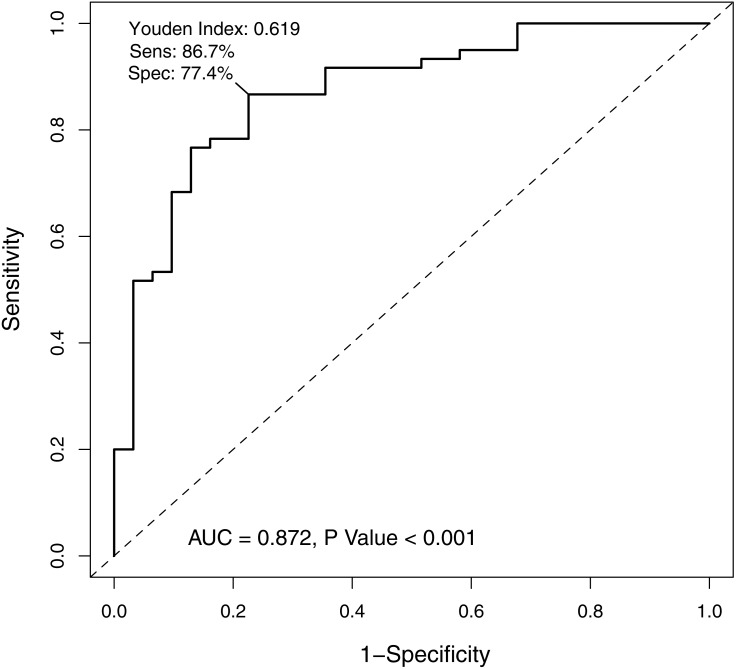
The receiver operating characteristic curve of the 16-gene signature. The area under the curve was 0.872 (*P* < 0.001), demonstrating that the 16-gene signature has high sensitivity and specificity for classification of oral tongue squamous cell carcinoma patients from the normal.

**Figure 4:**
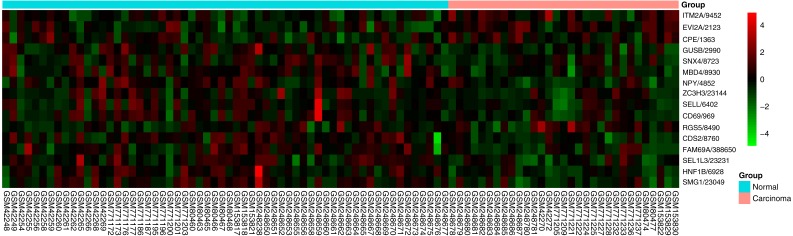
Heatmap of the 16-gene signature in five GEO datasets. The expression degrees are shown in different colors, from blue to orange with increasing expression.

In order to verify the classification reliability of the 16-gene signature, the multivariate logistic analysis was used to discriminate tongue carcinoma and normal samples in the combined GEO data sets. A ROC curve was generated, showing good sensitivity and specificity with average AUC of 0.872 (95% CI [0.795–0.949], *P* < 0.001) (Fig. 3). The signature came up with 86.7% prediction accuracy and 77.4% specificity at the Youden Index of 0.619. It meant that the 16-gene signature showed a good performance to classify the tongue carcinoma samples from the normal controls (Fig. 4). Also, 10-fold cross-validation showed the gene signature accuracy of 0.669 (95% CI [0.561–0.777], *P* < 0.001).

## Discussion

Cancer of the lip and oral cavity has caused great harm all over the world. In 2012, it brought 300,373 new cases and killed 145,353 people all around the world (Torre et al., 2015). In 2017, there were 16,400 estimated new cases and 2,400 estimated deaths in the United States (Siegel, Miller & Jemal, 2017). The current staging diagnosis, treatment choices and prognosis prediction of OTSCC are made primarily in line with the AJCC TNM staging system. However, when we enter the era of precision medicine, genetic analysis plays an increasingly important role in early molecular diagnosis, individualized treatment and accurate survival prediction (Ashley, 2015). Gene signatures have been proved to be valid in many cancers, such as colon cancer, kidney carcinoma and breast cancer (An et al., 2015; Bedognetti et al., 2015; Kanth et al., 2016; Xu et al., 2017; Zhan et al., 2015). However, there exist no studies with regard to gene signatures for tongue carcinoma.

In this study, we developed a 16-gene signature for patients with oral tongue squamous cell carcinoma based on TCGA and GEO data sets. Additionally, we exploited a risk score to classify OTSCC patients into high-risk and low-risk groups. As a result, the risk score was demonstrated to be an independent prognostic risk factor in the TCGA data set. The 16-gene signature was also proved to be effective to distinguish the carcinoma from normal samples in GEO data sets. The meaning of this study lied in the impact of 16-gene signature on prognosis for OTSCC patients. The 16-gene signature may be meaningful to illuminate the pathogenic mechanism of OTSCC. For all we know, it is the first study about gene signature for OTSCC patients.

All 16 genes from the signature were remarkably associated with the prognosis of OTSCC in our study. Of the 16 genes, HNF1B, NPY and SMG1 were found to be protective factors. Transcription factor HNF1B is a master regulator of gene expression, and loss of HNF1B may enhance cellular survival and exacerbate the development of chromophobe renal cell carcinomas (Sun et al., 2017). NPY, a neuropeptide abundantly produced by enteric neurons, is important in the regulation of intestinal inflammation, and the aberrant methylation of NPY is associated with intestinal tumor (Jeppsson, Srinivasan & Chandrasekharan, 2017; Roperch et al., 2013). SMG1 is suggested as a novel potential tumor suppressor gene in many cancers (Du et al., 2014). In regard to other risk factors, CD69 is expressed in several hemopoietic cells, and it takes part in cancer immunity. CD69 is correlated with poor clinical outcomes and is confirmed to be an independent prognosticator for blood system tumors (Del Poeta et al., 2012). CPE is a member of metallocarboxypeptidases family, and the CPE mRNA expression level can predict tumor recurrence in early-stage hepatocellular carcinoma, and predict poor prognosis in early-stage cervical cancer (Huang et al., 2016b; Shen et al., 2016). ITM2A is a poorly prognostic biomarker through inducing cell cycle arrest for ovarian cancer (Nguyen et al., 2016). RGS5 is highly expressed in malignant tumors, and overexpression of RGS5 promotes tumor metastasis by inducing epithelial-mesenchymal transition in hepatocellular carcinoma (Hu et al., 2013). SELL, also known as CD62L, is most expressed on urothelial carcinoma cells, and it is a potential marker predicting metastasis in patients with bladder cancer (Choudhary et al., 2015). The 16-gene signature was credible to illuminate the pathogenic mechanism of OTSCC. In addition, a functional enrichment analysis was conducted, however, no significant enrichment was detected when default parameters were chosen on DAVID or STRING. Perhaps it is because the functional study of these genes is not thorough at present.

Currently, microarrays and sequencing technologies are successful to identify new candidates in tumor biology (Dyrskjot et al., 2007). A strategy driven by data has been popular for gene signature search strategy by analyzing gene expression data set (Shi & He, 2016). A gene signature, which is composed of more than one gene, exhibits more excellently in prediction than a single biomarker. Therefore, gene signatures have been widely used in diagnostic analysis and prognostic prediction for plenty of diseases. For example, a risk score was developed based on the 6-gene signature and performed well in predicting overall survival for non-small cell lung cancer (Huang et al., 2016a). Genome-wide analysis of gene expression identified a 76-gene signature for patients with lymph-node-negative breast cancer, which could predict patients at high risk of distant recurrence powerfully (Wang et al., 2005). Combining two forms of artificial intelligence, neurofuzzy modeling and artificial neural networks, a prognostic gene signature was established, and the signature reflected a variety of carcinogenic pathways, recognizing tumor progression in non-muscle-invasive bladder cancer (Catto et al., 2010). As for our research, we got a prognostic 16-gene signature in OTSCC patients. Additionally, the signature could classify carcinoma and normal samples successfully.

In this study, we set up a 16-gene signature from two different platforms including GEO and TCGA data sets. Then we carried out a series of methods, including differentially expression identification as well as univariable and multivariable survival analysis, to screen target genes and calculate the risk score. Finally, the signature and risk score were substantiated. There are several limitations in our study. Firstly, we selected 16 genes just according to pure bioinformatics analysis. Further experiments are needed to validate the results based on carcinoma samples and clinical data. Secondly, the clinical information provided by the TCGA data set is not complete. Staging and grading information are missing for some patients, and data of treatment such as surgery, radiation or chemotherapy are absent. Thirdly, our signature was developed on the basis of only 91 patients from GEO data sets and 101 patients from the TCGA data set. We plan to better incorporate more data sets to confirm the results in the future work. To improve these, we plan to collect tumor samples as well as clinical prognostic information and prove the results through experiments. Since functional studies of these 16 genes are limited now, more researches about their functional links and substantial association with patient survival are necessary and meaningful.

## Conclusions

In conclusion, our results demonstrated that the 16-gene signature might serve as a predictor for prognosis of OTSCC patients, which could be applied to clinical practice effectively. Further studies are necessary to confirm the findings in the future.

##  Supplemental Information

10.7717/peerj.4062/supp-1 Supplemental Information 1 Raw data and code

10.7717/peerj.4062/supp-2 Table S1 GO biological process enrichment analyses of differentially expressed genes for oral tongue squamous cell carcinoma Note: GO, Gene Ontology.

10.7717/peerj.4062/supp-3 Table S2 KEGG enrichment analyses of differentially expressed genes for oral tongue squamous cell carcinoma Note: KEGG, Kyoto Encyclopedia of Genes and Genomes.

10.7717/peerj.4062/supp-4 Table S3 Three significant modules in the protein-protein interaction network

10.7717/peerj.4062/supp-5 Figure S1 Protein-protein interaction network of differentially expressed genes for five GEO datasets

10.7717/peerj.4062/supp-6 Figure S2 The calibration curves predicting overall survival at (a) 3-year and (b) 5-year Risk score-predicted OS rates are on the *x*-axis, and actual survival rates are on the *y*-axis. The gray line is the ideal prediction for each plot. OS, overall survival.

## Additional Information and Declarations

### Data Availability

The following information was supplied regarding data availability: The raw data and code have been provided in a Supplemental File.
