# Supplementary material for: A 16-gene signature predicting prognosis of patients with oral tongue squamous cell carcinoma
Source: PeerJ. 2017 Nov 17;5:e4062. doi: 10.7717/peerj.4062 (PMC5695251; doi:10.7717/peerj.4062)
Supplement: Table S1 — Note: GO, Gene Ontology. [file peerj-05-4062-s002.docx]

**Supplementary Table 1. GO biological process enrichment analyses of differentially expressed genes for oral tongue squamous cell carcinoma.**

| **GO Term** | **Count** | ***P* Value** |
| --- | --- | --- |
| GO:0006614~SRP-dependent cotranslational protein targeting to membrane | 28 | 4.86×10^-26^ |
| GO:0019083~viral transcription | 28 | 8.62×10^-24^ |
| GO:0006413~translational initiation | 30 | 9.63×10^-24^ |
| GO:0000184~nuclear-transcribed mRNA catabolic process, nonsense-mediated decay | 28 | 4.94×10^-23^ |
| GO:0006364~rRNA processing | 30 | 4.01×10^-18^ |

Note: GO, Gene Ontology.
