# Supplementary material for: A 16-gene signature predicting prognosis of patients with oral tongue squamous cell carcinoma
Source: PeerJ. 2017 Nov 17;5:e4062. doi: 10.7717/peerj.4062 (PMC5695251; doi:10.7717/peerj.4062)
Supplement: Table S2 — Note: KEGG, Kyoto Encyclopedia of Genes and Genomes. [file peerj-05-4062-s003.docx]

**Supplementary Table 2.** **KEGG enrichment analyses of differentially expressed genes for oral tongue squamous cell carcinoma.**

| **KEGG Term** | **Count** | ***P* Value** |
| --- | --- | --- |
| hsa03010:Ribosome | 28 | 1.58×10^-17^ |
| hsa05416:Viral myocarditis | 7 | 2.85×10^-03^ |
| hsa03060:Protein export | 4 | 1.89×10^-02^ |
| hsa04142:Lysosome | 8 | 3.18×10^-02^ |
| hsa04650:Natural killer cell mediated cytotoxicity | 8 | 3.30×10^-02^ |

Note: KEGG, Kyoto Encyclopedia of Genes and Genomes.
