# Supplementary material for: A 16-gene signature predicting prognosis of patients with oral tongue squamous cell carcinoma
Source: PeerJ. 2017 Nov 17;5:e4062. doi: 10.7717/peerj.4062 (PMC5695251; doi:10.7717/peerj.4062)
Supplement: Table S3 [file peerj-05-4062-s004.docx]

**Supplementary Table 3. Three significant modules in the protein-protein interaction network.**

| **Modules** | **Composition** |
| --- | --- |
| Module A | RPL17, NACA, RPL19, RPL15, RPL35, RPS3, RPS25, RPL32, RPS3A, RPL34, RPL3, TPT1, BTF3, RPL5, RPL4, RPL10A, RPL12, RPS23, RPS27A, NSA2, EEF1A1, SRP54, MRPL3, SEC11A, SMG1, RPL24, RPL23A, RPS4X, RPS6, FBL, RPS16, RPL41, RPL22, RPL21, EIF4H, EIF4A2, RPS12, RPS13, RPS11 |
| Module B | HNRNPH2, DDX46, DNAJC8, SNRNP200, HNRNPA2B1, SRSF11, HNRNPA1, SF3A1 |
| Module C | CD48, CD83, PLCB3, CCK, NPY, CXCR4, CD69, SELL, P2RY14, EDN2, CCL19, TRH, CCL4 |
