# Supplementary figures and images for: A 16-gene signature predicting prognosis of patients with oral tongue squamous cell carcinoma
Source: PeerJ. 2017 Nov 17;5:e4062. doi: 10.7717/peerj.4062 (PMC5695251; doi:10.7717/peerj.4062)

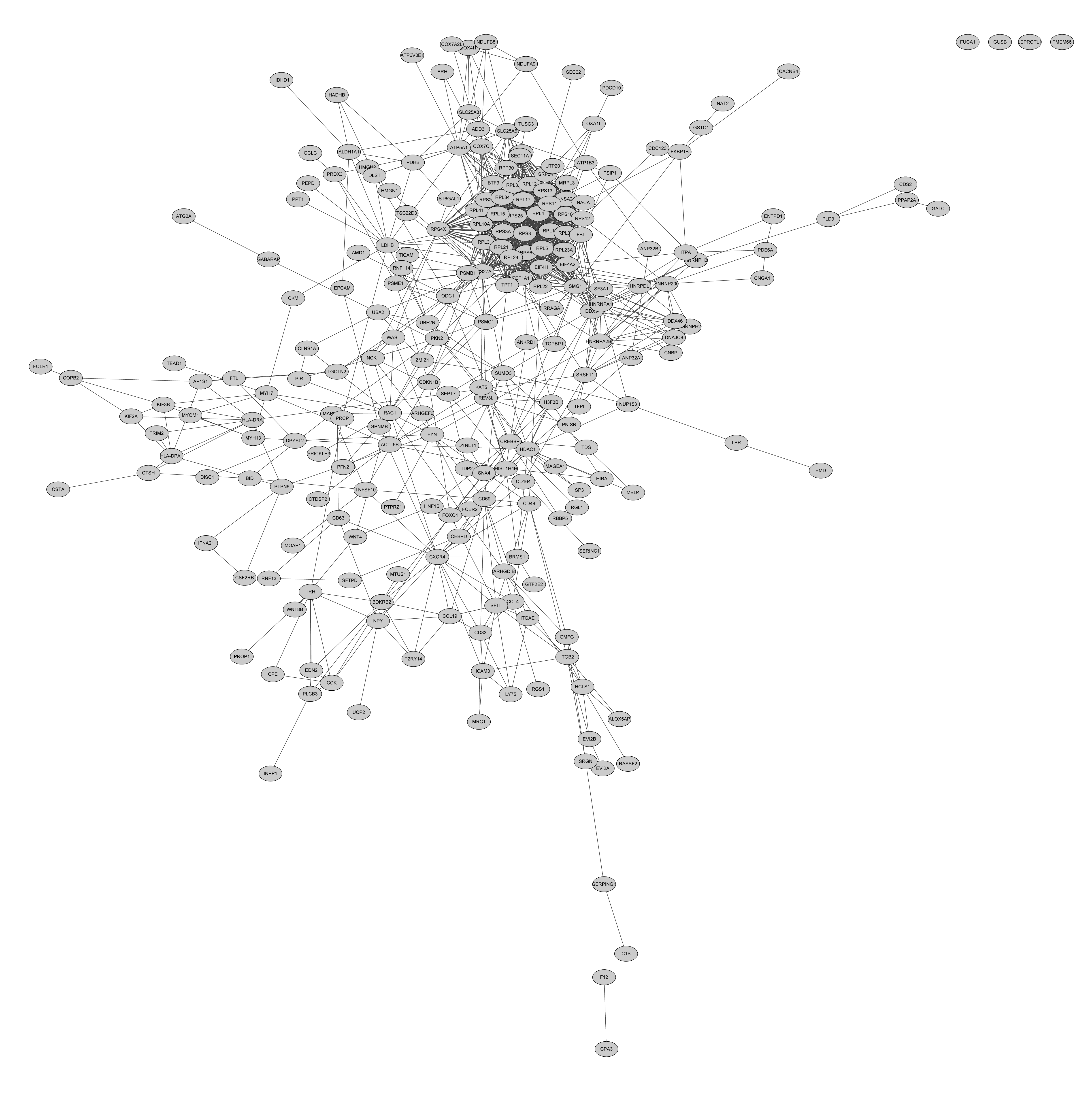

Supplement: Figure S1 [file peerj-05-4062-s005.png]

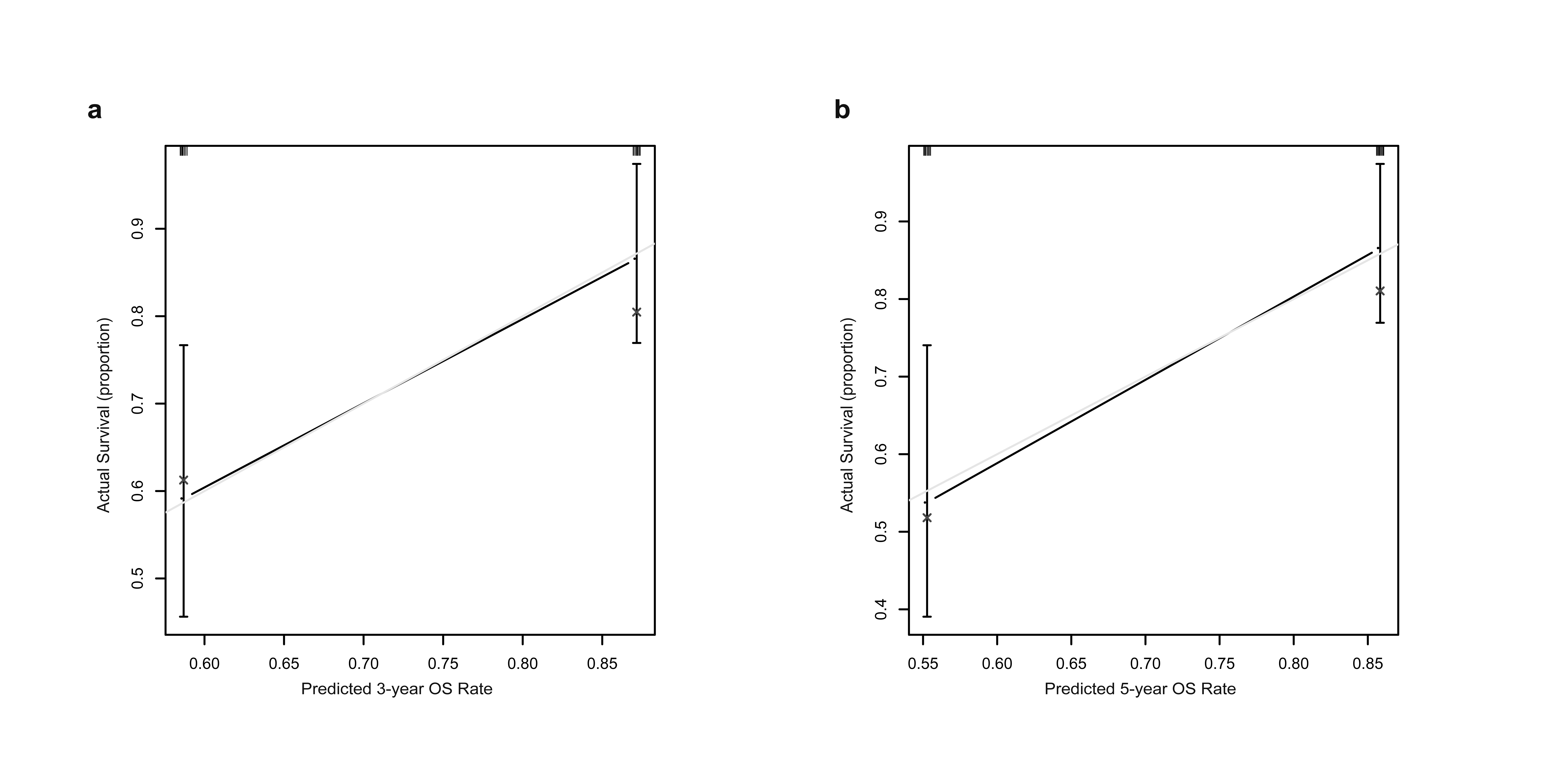

Supplement: Figure S2 — Risk score-predicted OS rates are on the x-axis, and actual survival rates are on the y-axis. The gray line is the ideal prediction for each plot. OS, overall survival. [file peerj-05-4062-s006.png]
